# Nipple Discharge of CA15-3, CA125, CEA and TSGF as a New Biomarker Panel for Breast Cancer

**DOI:** 10.3390/ijms15069546

**Published:** 2014-05-28

**Authors:** Gangping Wang, Yan Qin, Junxi Zhang, Jinhui Zhao, Yun’ai Liang, Zuofeng Zhang, Meihua Qin, Yanqing Sun

**Affiliations:** 1 Department of Pathology, Rizhao People’s Hospital, Affiliated Rizhao People’s Hospital of Jining Medical College, 126 Taian Road, Rizhao 276826, China; E-Mail: rzlyunai@163.com; 2 Department of Clinical Laboratory, Rizhao People’s Hospital, Rizhao 276826, China; E-Mails: tongshuoshuo@163.com (Y.Q.); qmhrzph@sina.com (M.Q.); wgprzph93@sina.com (Y.S.); 3 Department of General Surgery, Rizhao People’s Hospital, Rizhao 276826, China; E-Mails: lfhrzph@163.com (J.Z.); zzf625@163.com (Z.Z.); 4 Department of Gynaecology, Zhucheng People’s Hospital, Zhucheng 262200, China; E-Mail: zhanghong722@163.com

**Keywords:** breast cancer, nipple discharge, serological biomarker, cancer antigen 15-3, cancer antigen 125, carcinoembryonic antigen, malignant tumor-specific growth factor

## Abstract

Breast cancer is the second leading cause of cancer death in women. Serum biomarkers such as cancer antigen 15-3 (CA15-3), cancer antigen 125 (CA125), and carcinoembryonic antigen (CEA) can be used as diagnostic and prognostic factors and can also provide valuable information during follow-up. However, serum protein biomarkers show limited diagnostic sensitivity and specificity in stand-alone assays because their levels reflect tumor burden. To validate whether biomarkers in nipple discharge may serve as novel biomarkers for breast cancer, we composed a panel of potential cancer biomarkers, including CA15-3, CA125, CEA, and malignant tumor-specific growth factor (TSGF), and evaluated their expression in both serum and nipple discharge in order to explore the expression and significance of estrogen receptor (ER), progestrone receptor (PR), epidermal growth factor receptor type 2 (HER2/neu), CA15-3, CA125, CEA, and TSGF expression for their combined predictive value for breast cancer and in judging the prognosis of breast cancer. Univariate analysis revealed that combined detection of CA15-3, CA125, CEA, and TSGF in nipple discharge served as novel biomarkers for the diagnosis and prognosis of breast cancer, but in the multivariate analyses the adverse effects of the four biomarkers combination in nipple discharge positivity on overall survival were lost. Multivariate analysis revealed that the positivity of the combined detection of the four biomarkers in both nipple discharge and serum was significantly higher than that of other detection methods. Thus, the combined detection of these four biomarkers both in serum and nipple discharge was retained as an independent prognostic variable in breast cancer patients. Our results indicate that CA15-3, CA125, CEA, and TSGF in nipple discharge can serve as novel biomarkers in the diagnosis and prognosis of breast cancer.

## 1. Introduction

Breast cancer is the most common form of cancer in women and the second leading cause of death due to malignant disease in females after lung cancer [1,2]. In recent years, the incidence of breast cancer has significantly increased in both Eastern and Western countries. As a result, numerous studies worldwide have sought to determine the most effective ways to treat breast cancer, assess the rapeutic effects, correctly evaluate prognosis, and identify postoperative recurrence in patients [3,4,5]. Mammography, the current routine method used for early detection, has shown limited sensitivity for the detection of tumors in dense breast tissue. However, blood tests are easy to perform and would omit the imaging-related problem of high breast density [6]. Additionally, blood testing would provide a greater benefit in younger women, who are now excluded from most breast cancer screening programs mainly because the prevalence of dense breast tissue is very high in this group [1]. Components of bodily fluids are ideal biomarkers for disease diagnosis because of their simplicity of detection. For example, the serum has long been considered a rich source of biomarkers, and several serum cancer biomarkers, such as cancer antigen 125 (CA125), carcinoembryonic antigen (CEA), and cancer antigen 15-3 (CA15-3), have been proposed [2,7,8,9]. However, serum protein biomarkers are often not sufficiently sensitive to be used for screening and early diagnosis because their levels reflect tumor burden [10]. Unlike traditional tumor markers, nipple discharge autoantibodies against tumor antigens are detectable even when the tumor is very small, making them potential biomarkers for early cancer diagnosis [7,11]. Moreover, the advantages of simple detection of nipple discharge components using target antigens and secondary reagents, in contrast to serum protein markers, whose detection requires two different monoclonal antibodies, make it easy to construct a multiplex tumor-associated autoantibody assay. However, the few available biomarkers that are used for the early detection of breast cancer show limited diagnostic sensitivity and specificity in stand-alone assays [12,13,14]. Thus, a panel of biomarkers may show better predictive performance than individual markers. Moreover, because breast cancer is a heterogeneous disease, it may require a panel of several biomarkers to allow the detection of different subtypes [15]. Combinations of biomarkers have not been extensively tested for their usefulness in early breast cancer detection [8,16,17], although several studies have investigated the performance of a panel of cancer biomarkers compared with individual markers alone [18,19,20,21].

We composed a panel of potential cancer biomarkers for breast cancer to study their expression and clinical significance in the diagnosis and treatment of breast cancer. Two serum proteins that have been approved by the Food and Drug Administration (FDA) as biomarkers to monitor chemotherapy in patients with advanced breast cancer were included: CA15-3 and CEA [14]. In addition, the tumor marker CA125 is primarily used together with transvaginal ultrasound for the early detection of ovarian cancer in women with hereditary syndromes [14], but has also been suggested as a tumor marker for breast cancer [22]. Regarding the serum markers CA15-3, CEA, and CA125, a relationship with breast cancer was reported in previous studies, although each marker alone was not sufficiently discriminative. We also included another cancer antigen, malignant tumor-specific growth factor (TSGF), which was recently shown to be differentially expressed in the serum of breast cancer and control subjects.

The present study investigated the value of a panel of four potential serum/nipple discharge markers in the diagnosis and prognosis of breast cancer. In this study, the follow-up duration of serum biomarkers ranged from 3 months to 2 years, and the levels of the above indicators were detected through dynamic blood draw samples. The expression and significance of the combined indicators CA15-3, CA125, CEA, and TSGF were evaluated in serum (*n* = 256) and nipple discharge (*n* = 86) obtained from Chinese females at Rizhao People’s Hospital between June 2006 and June 2013. In addition, we measured the expression of the estrogen receptor (ER), progesterone receptor (PR), and HER2/neu and the Ki-67 proliferation index to further assess the prognosis of breast cancer patients. A receiver operating characteristic (ROC) curve was constructed, and the area under the curve (AUC) was calculated using each algorithm. We then extracted a classifier consisting of a subset of markers yielding the best classification performance in the test sets. ROC curves were generated based on the comparison with the control group. The cut-off values were selected using ROC curves as the reference, and the Youden’s index estimated probability at the maximum sum of the sensitivity and specificity was used for the CA15-3, TSGF, CA125, and CEA serum and nipple discharge marker cut-off values at the clinical laboratory of Rizhao People’s Hospital. Single marker detection positivity was defined as a biomarker level greater than the cut-off value, whereas combined detection positivity was defined as one of the biomarkers showing positivity in the serum and one of biomarkers showing positivity in nipple discharge, or more than one biomarker in the serum and/or nipple discharge showing positivity. Clinical and pathological information documented at the time of surgery included the clinical stage of the cancer, grade and histology of the tumor, and amount of the remaining tumor. Menopausal status was documented, and the response to chemotherapy was monitored. Tumors were staged according to the American Joint Committee on Cancer criteria [23]. Histologic classification was based on the World Health Organization (WHO) and the modified Bloom-Richardson score. Patients were monitored for survival and disease progression (no apparent progression or progression) for a median duration of 68 months (range, 3–78 months), and patients were censored if the follow-up period was less than 6 months. Follow-up information was available for 86 of the patients. Seventeen (19.8%) of these patients relapsed, and nine (10.5%) died during the course of the follow-up period. Sixty cases of normal pregnant women with no evidence of malignancy or familial history of breast cancer constituted the control group.

Univariate analysis revealed that that combined detection of CA15-3, CA125, CEA, and TSGF in nipple discharge served as novel biomarkers in the diagnosis and prognosis of breast cancer. Multivariate analysis revealed that the positivity of the combined detection of the four biomarkers both in serum and nipple discharge was significantly higher than the positivity of other detection methods. Thus, the panel containing these four biomarkers both in serum and nipple discharge was retained as an independent prognostic variable in breast cancer patients. Our results indicate that these biomarkers may permit earlier detection of breast cancer as well as earlier outcome prediction for patients, and this new approach will likely contribute to the development of personalized therapy for breast cancer.

## 2. Results and Discussion

### 2.1. The Levels of Biomarkers in the Serum and Nipple Discharge in Three Patient Groups

**Figure 1:**
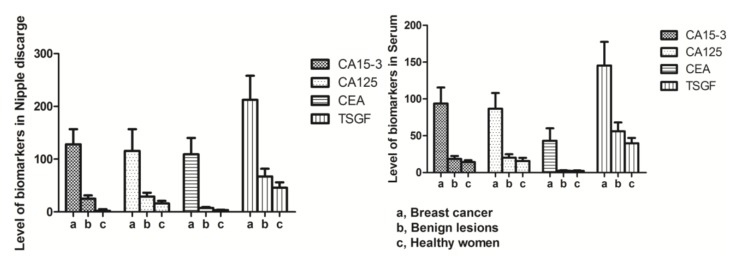
Nipple-discharge and serum biomarkers levels among the three groups.

The nipple discharge and serum levels of CA15-3, CA125, CEA, and TSGF in the observation breast cancer group were all significantly higher than those in patients with benign breast lesions (CA15-3: *t* = 24.44 and 10.43; CA125: *t* = 13.71 and 20.22; CEA: *t* = 21.56 and 22.44; TSGF: *t* = 20.51 and 17.88, respectively; all *p* < 0.01) and healthy pregnant women (CA15-3: *t* = 25.65 and 24.51; CA125: *t* = 20.22 and 21.17; CEA: *t* = 22.42 and 15.97; TSGF: *t* = 23.07 and 20.81, respectively; all *p* < 0.01). The levels of the four biomarkers in nipple discharge were significantly higher than those in the serum in the observation breast cancer group (*t* = 6.35, 4.11, 12.28, and 7.86, respectively; all *p* < 0.01) (Table 1 and Figure 1). The nipple aspirate fluid mirrored the ductal-lobular microenvironment, and the levels of detection of the four biomarkers in nipple discharge were useful for analysis of the ductal-lobular microenvironment in breast disease. The intraductal approach for protein and proteomic analyses may provide a panel of biomarkers to strengthen the armory against breast cancer [7]. In Mannello’s report, the analysis of nipple aspirate fluid (mirroring the ductal-lobular microenvironment) was a useful tool for the analysis of metabolic pathways within the mammary gland, deepening the knowledge of the biomolecular mechanisms of breast cancer initiation and progression [7]. Protein and proteomic high-throughput technologies can provide the polypeptide signatures of nipple aspirate fluid, a breast secretion collected noninvasively from healthy individuals and cancer patients, and this fluid is useful for diagnostic measures because breast cancer develops from the ductal-lobular epithelium. Many biomarkers have been evaluated in breast diseases, including CA15-3, CA125, CEA, and TSGF. Among them, CA15-3 and CEA are the most commonly used. CA15-3, a variant of mammary epithelial surface glycoprotein and an antigen commonly related to breast cancer, is considered an indicator for detecting the recurrence and metastasis of carcinoma. Studies have also confirmed that, as a glycoprotein, CA125 expression can be identified using a monoclonal antibody, and this expression shows a correlation with the genesis and development of breast cancer. In comparison, CEA, as a broad-spectrum tumor marker, is used to detect the postoperative recurrence rate of various epithelial tumors and determine patient prognosis. If a cancerous tumor cell growing out of control is present in the breast, the levels of CA15-3, CA125, CEA, and TSGF may increase as the number of cancer cells increases. In our study, the levels of the four biomarkers in nipple discharge were significantly higher than those in serum (*p* < 0.01). Additionally, the nipple discharge and serum levels of CA15-3, TSGF, CA125, and CEA in breast carcinoma were significantly higher than those in benign breast lesions (*p* < 0.01). In Mannello’s report, the different expression levels of major nipple aspirate fluid proteins, separated using 1D polyacrylamide gels, were shown to be valuable for the early detection of breast cancer in women with increased risk. However, the failure to recognize a single marker with sufficient clinical sensitivity and/or specificity has driven the identification of multiple breast cancer marker proteins by 2D electrophoresis [7]. In addition, mass spectrometry-based proteomic approaches (surface enhanced laser desorption ionization-proteinchip and matrix assisted laser desorption ionization/time of flight technologies, SELDI- and MALDI-TOF technologies) have allowed the characterization of differential nipple aspirate fluid proteomic fingerprints between healthy individuals and breast cancer patients [7]. Of note, the few biomarkers that have been tested for the early detection of breast cancer have shown limited diagnostic sensitivity and specificity in stand-alone assays [12].

**Table 1:** Comparison of serum and nipple discharge biomarkers levels among the three groups (mean ± SD).

Group	*n*	CA15-3 (U/mL)	CA125 (U/mL)	CEA (ng/mL)	TSGF (U/mL)
Nipple discharge					
Breast cancer	86	128.21 ± 28.63	115.71 ± 41.08	109.23 ± 30.94	212.42 ± 45.71
Benign lesions	136	25.13 ± 6.14 ^a^	29.41 ± 7.22 ^a^	7.46 ± 1.75 ^a^	67.01 ± 14.98 ^a^
Healthy women	60	15.95 ± 3.23 ^b^	16.11 ± 4.76 ^b^	3.28 ± 0.87 ^b^	45.78 ± 10.36 ^b^
Serum					
Breast cancer	86	93.79 ± 21.80	86.68 ± 21.37	43.29 ± 16.81	145.46 ± 32.11
Benign lesions	136	18.61 ± 3.98 ^c^	20.29 ± 4.60 ^c^	2.40 ± 0.72 ^c^	56.01 ± 11.99 ^c^
Healthy women	60	14.22 ± 2.47 ^d^	15.76 ± 4.25 ^d^	2.33 ± 0.54 ^d^	39.78 ± 7.36 ^d^

CA15-3: cancer antigen 15-3; CEA: carcinoembryonic antigen; CA-125: cancer antigen 125; TSGF: malignant tumor-specific growth factor; Nipple discharge: ^a^ *p* < 0.01 between benign and breast cancer patients; ^b^ *p* < 0.01 between healthy control and breastcancer patients; Serum: ^c^ *p* < 0.01 between benign and breast cancer patients; ^d^ *p* < 0.01 between healthy control and breastcancer patients.

### 2.2. Discriminative Diagnostic Value of Biomarkers in the Nipple Discharge and Serum of Breast Cancer Patients

**Figure 2:**
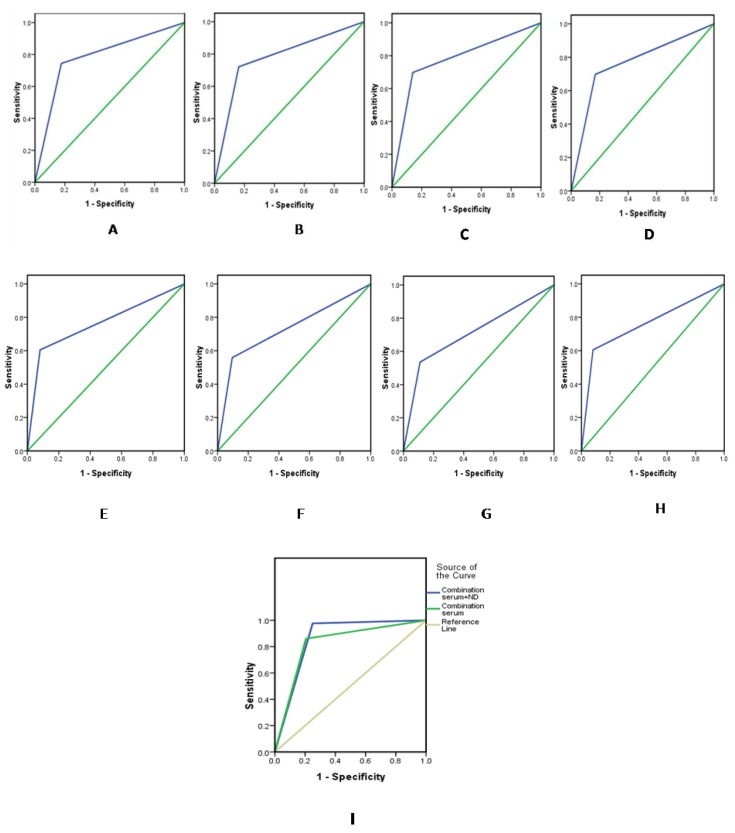
The discriminatory values, according to the area under the curve (AUC) values, for nipple discharge (**A**–**D**) and serum (**E**–**H**). (**A**) CA15-3; (**B**) CA125; (**C**) CEA; (**D**) TSGF; (**E**) CA15-3; (**F**) CA125; (**G**) CEA; (**H**) TSGF; and (**I**) Combination (nipple discharge + serum).

In the present study, the follow-up duration of serum biomarkers ranged from 3 months to 2 years, and the levels of the above indicators were detected using dynamic blood draw samples. At the clinical laboratory of Rizhao People’s Hospital, the cut-off values for CA15-3, TSGF, CA125, and CEA in the serum were 25.00, 70.00, 35.00 U/mL, and 3.40 ng/mL, respectively, and those in the nipple discharge were 35.00, 95.00, 40.00 U/mL, and 9.8 ng/mL, respectively. The discriminative values of CA15-3, TSGF, CA125, and CEA in nipple discharge and serum and those for their combined detectionin terms of breast cancer diagnosis are shown in Table 2. ROC curves were constructed, and AUCs were calculated using each algorithm shown in Figure 2 and Table 3. The sensitivity of the four serum tumor markers in combination was only 80.2%. By contrast, the combined detection in both nipple discharge and serum was 97.7%, and the negative predictive value was 99.0%. The sensitivity of combined detection in both nipple discharge and serum was significantly higher than that of the individual markers and other combination detection methods (*p* < 0.05). Serum has long been considered a rich source of biomarkers, and CA15-3, TSGF, CA125, and CEA have been proposed as serum-based biomarkers. However, serum protein biomarkers are often not sufficiently sensitive for screening and early diagnosis because their levels reflect tumor burden. Unlike traditional tumor markers, nipple discharge autoantibodies against tumor antigens are detectable even when the tumor is very small, which makes them potential biomarkers for early cancer diagnosis. Nipple discharge is common, accounting for 5% of all breast-related symptoms [24], and abnormal discharge is most commonly caused by benign conditions such as intraductal papillomas, duct ectasia, papillomatosis, mastitis, and fibrocystic changes [11]. The incidence of breast cancer in patients presenting with abnormal nipple discharge is between 6% and 21% [25]. Most patients with breast cancer who manifest isolated discharge show early stage disease associated with ductal carcinoma *in situ* (DCIS). Discharge due to DCIS has been shown to be a marker of extensive disease, which often requires mastectomy to achieve adequate surgical margins [26,27]. Diffusely spreading intraductal carcinomas, which often show no clinically palpable breast mass, can manifest as pathological discharge. Moreover, the advantages of simple detection with nipple discharge using a target antigen and secondary reagents, in contrast to tissue biopsy and traumatic examination, enable the use of a multiplex tumor-associated autoantibody assay. However, the few biomarkers that have been tested for the early detection of breast cancer show limited diagnostic sensitivity and specificity in stand-alone assays [12], although a panel of biomarkers may show better predictive performance than individual markers. In addition, because breast cancer is a heterogeneous disease, it may require a panel of several biomarkers to allow the detection of different subtypes. Hence, the combined detection of tumor markers in both nipple discharge and serum represents a promising approach for the detection and treatment of patients with breast cancer, as well as the determination of progression and prognosis of the disease. In the present study, univariate analysis revealed that a panel of CA15-3, CA125, CEA, and TSGF in nipple discharge represent novel biomarkers for the diagnosis of breast cancer. Multivariate analysis revealed that the positivity of these four biomarkers used for combined detection was significantly higher than the positivity of other detection methods, and this panel was retained as an independent variable in breast cancer patients. Together, our results indicate that these biomarkers may permit the earlier detection of breast cancer.

**Table 2:** The diagnostic discriminative value of the four biomarkers in breast cancer (%).

Groups	Sensitivity	Accuracy	Specificity	PV+	PV−
Nipple discharge					
CA15-3	74.4	80.5	82.4	57.1	91.1
CA125	72.1	81.0	83.8	58.5	90.5
CEA	69.8	82.2	86.0	61.2	90.0
TSGF	69.8	79.9	83.1	56.6	86.7
Serum					
CA15-3	60.5	84.4	91.9	70.3	88.0
CA125	55.8	82.2	90.4	64.9	88.6
CEA	53.5	80.5	89.0	60.5	85.8
TSGF	62.8	84.4	91.2	69.3	88.6
Combination	97.7 *	80.5	75.0	55.3	99.0 *

* Compared with other items, *p* < 0.01; CA15-3: cancer antigen 15-3; CEA: carcinoembryonic antigen; CA125: cancer antigen 125; TSGF: malignant tumor-specific growth factor; PV+: positive predictive value; PV−: negative predictive value; Combination: combined with a panel of the four serum/nippledischarge markers; combined detection positivity was defined as positivity of one of the biomarkers in serum and positivity of one of the biomarkers in nipple discharge, or positivity of more than one biomarker in nipple discharge, or positivity of more than one biomarker in serum and positivity of more than one biomarker in nipple discharge.

A previous study by Kim *et al.* showed that breast cancer markers with low sensitivity individually showed increased sensitivity for the diagnosis of breast cancer when used in combination [19]. In our study, the panel of four serum/nipple-discharge markers combined showed the highest AUC value (0.863) and exhibited a diagnostic accuracy of 80.5%, sensitivity of 97.7%, and specificity of 75.0%. The AUC value and diagnostic sensitivity were significantly higher when combined in comparison to the four markers in serum, in nipple-discharge or used in isolation.

Unlike traditional serum tumor markers, nipple discharge autoantibodies against tumor antigens are detectable even when the tumor is very small, which makes them potential biomarkers for early cancer diagnosis. Moreover, the advantages of simple detection using nipple discharge with target antigens and secondary reagents, in contrast to tissue biopsy and traumatic examination, enable the construction of multiplex tumor-associated autoantibody assays. However, the few biomarkers that have been tested for the early detection of breast cancer show limited diagnostic sensitivity and specificity in stand-alone assays [12]. Thus, a panel of biomarkers may provide better predictive performance than individual markers. In addition, because breast cancer is a heterogeneous disease, it may require a panel of several biomarkers to allow the detection of different subtypes. Hence, the combined detection of tumor markers in both nipple discharge and serum may hold promise for the discovery and treatment of new breast cancer cases.

**Table 3:** ROC curve analysis of clinicopathological data.

Parameter	AUC	95% CI		*p* value
		Lower bound	Upper bound	
Nipple discharge				
CA153 (U/mL)	0.784	0.719	0.849	<0.01
TSGF (U/mL)	0.764	0.697	0.842	<0.01
CA125 (U/mL)	0.780	0.713	0.846	<0.01
CEA (ng/mL)	0.779	0.712	0.846	<0.01
Combination ND	0.827	0.769	0.885	<0.01
Serum				
CA153 (U/mL)	0.762	0.692	0.832	<0.01
TSGF (U/mL)	0.770	0.701	0.839	<0.01
CA125 (U/mL)	0.732	0.659	0.804	<0.01
CEA (ng/mL)	0.712	0.639	0.786	<0.01
Combination ND + Serum	0.863	0.814	0.913	<0.01

CA15-3: cancer antigen 15-3; CEA: carcinoembryonic antigen; CA-125: cancer antigen 125; TSGF: malignant tumor-specific growth factor; ROC: receiver operating characteristic; AUC: area under the curve; CI: confidence interval; ND: nipple discharge.

### 2.3. The Relationship between the Nipple Discharge Levels of Tumor Markers and Biological Parameters within a Breast Cancer Group

Various clinical and pathological factors were analyzed and compared to the nipple discharge concentrations of the four biomarkers. The impact of clinicopathological parameters on the nipple discharge levels of tumor markers within the study groups is shown in Table 4. Breast cancer tumor size, onset age, and menarcheand menopause age showed no significant association with the nipple discharge level of the four biomarkers among the breast cancer groups. Based on body mass index (BMI), the female patients were categorized into two groups: those with a BMI below 30 kg/m^2^ or greater than or equal to 30 kg/m^2^. No significant association was found between the four biomarker levels in nipple discharge and BMI. However, the nipple discharge levels of the four biomarkers were markedly higher in ER- and PR-negative patients compared to ER- and PR-positive patients. Additionally, the serum/nipple discharge concentrations of the four biomarkers were influenced by factors such as vascular endothelial growth factor (VEGF) expression and the Ki-67 proliferation index in tumor tissue. VEGF was localized in the cytoplasm and the membrane. Cells were classified according to the positive rate and color intensity as follows: negative, number of positive cells <25%; positive, brown particles, number of positive cells ≥25%. VEGF, which promotes angiogenesis and promotes the proliferation of endothelial cells, also exerts an important effect in the genesis, development, metastasis, and recurrence of various tumors. Due to the intimate association between the genesis, development, metastasis, and infiltration of breast cancer, the serum/nipple discharge levels represent an important indicator of metastasis and infiltration of breast cancer in the clinical setting. In fact, a significant difference was noted in the serum/nipple discharge levels of tumor markers between HER2/neu-positive patients and HER2/neu-negative patients and between patients with a Ki-67 proliferation index ≤14% and patients with a Ki-67 proliferation index >14% (*p* < 0.05). Furthermore, with an increase in pathological staging, the levels of the four biomarkers in serum (data not shown) and nipple discharge gradually increased (*p* < 0.01). In addition, the biomarker levels in serum (data not shown) and nipple discharge were significantly increased in patients with lymph node metastasis and distal metastasis compared to patients without lymph node metastasis or distal metastasis (*p* < 0.01). The biomarker levels in cases of distal metastasis were also notably higher than those in cases of regional lymph node metastasis (*p* < 0.01). The postoperative follow-up results revealed that the levels of nipple discharge and serum CA15-3, CA125, CEA, and TSGF in the recurrence group were clearly higher than those in the non-recurrence group (*p* < 0.01) (Table 4). During the follow-up period, which ranged from 3 to 78 months, there were 17 recurrent patients. Our results indicate that the levels of CA15-3, TSGF, CA125, and CEA in VEGF-positive patients with breast cancer were significantly higher than those in VEGF-negative patients. The high nipple discharge levels of the four biomarkers in breast carcinoma patients also showed a positive correlation with poor differentiation, high Ki-67 proliferation index (>14%), advanced grade and clinical stage, lymph node metastasis, and tumor recurrence (*p <* 0.05), but a negative correlation with the level of ER and PR expression.

**Table 4:** The impact of various parameters on nipple discharge biomarker levels in the breast cancer group (*n* = 86).

Subgroup	*n*	CA15-3 (U/mL) mean ± SD	CA125 (U/mL) mean ± SD	CEA (ng/mL) mean ± SD	TSGF (U/mL) mean ± SD
Tumor size					
≤2 cm	54	126.48 ± 23.34	113.96 ± 27.58	108.84 ± 25.12	206.78 ± 36.67
>2 cm	32	129.03 ± 28.37	116.89 ± 40.98	111.13 ± 32.84	214.89 ± 39.95
Age					
≥50 years	58	129.01 ± 30.54	116.37 ± 41.24	112.94 ± 31.06	215.78 ± 38.91
<50 years	28	127.72 ± 29.66	76.54 ± 22.43	107.01 ± 28.38	209.67 ± 35.53
Age at Menarche					
≤14 years	60	125.28 ± 25.69	113.65 ± 30.23	111.24 ± 29.33	210.83 ± 35.98
>14 years	26	130.06 ± 31.77	116.89 ± 41.01	108.20 ± 28.27	213.77 ± 37.93
Age at Menopause					
≤50 years	69	136.61 ± 33.39	145.17 ± 51.72	131.42 ± 41.88	233.43 ± 41.23
>50 years	17	115.89 ± 18.25	112.57 ± 39.53	99.91 ± 28.39	198.87 ± 32.56
BMI					
<30 kg/m^2^	62	129.74 ± 31.64	117.01 ± 42.75	107.91 ± 28.91	215.72 ± 38.21
≥30 kg/m^2^	24	127.51 ± 29.36	112.09 ± 30.01	112.62 ± 29.47	210.65 ± 34.37
ER & PR					
positive	72	129.46 ± 22.98	119.98 ± 28.87	92.62 ± 29.89	189.23 ± 32.31
negative	14	157.83 ± 37.56	121.38 ± 30.32	128.98 ± 31.11	251.71 ± 53.89
HER2/neu					
positive	28	151.43 ± 32.42	120.69 ± 31.37	117.34 ± 29.65	243.56 ± 54.76
negative	58	125.74 ± 26.05	98.55 ± 20.79	85.62 ± 27.47	187.34 ± 33.96
VEGF					
positive	59	147.62 ± 41.23	111.42 ± 31.88	123.33 ± 32.46	242.45 ± 43.88
negative	27	112.28 ± 26.72	80.99 ± 22.39	104.44 ± 25.12	189.79 ± 31.96
Ki-67					
>14%	21	165.24 ± 39.33	139.19 ± 44.32	125.17 ± 33.42	237.78 ± 46.53
≤14%	65	110.36 ± 25.78	106.35 ± 25.17	103.56 ± 25.67	201.78 ± 31.56
Grade					
III	33	312.56 ± 66.13	165.31 ± 63.42	195.67 ± 56.32	263.21 ± 68.43
II	53	68.20 ± 24.35	96.42 ± 26.34	78.32 ± 14.69	176.42 ± 23.69
Lymph node metastasis					
positive	42	128.21 ± 28.63	115.71 ± 41.08	109.23 ± 30.94	212.42 ± 45.71
negative	44	20.01 ± 10.97	21.43 ± 7.83	5.49 ± 2.78	46.891 ± 16.77
Distal metastasis					
positive	17	572.23 ± 105.25	198.62 ± 47.24	158.23 ± 55.24	228.22 ± 73.15
negative	69	90.64 ± 23.33	143.63 ± 26.02	105.01 ± 28.32	189.63 ± 26.31
Recurrence					
positive	17	176.32 ± 105.25	189.62 ± 98.78	171.77 ± 56.76	236.65 ± 98.76
negative	69	107.43 ± 24.68	101.34 ± 89.34	87.62 ± 23.19	176.76 ± 32.34

CA15-3: cancer antigen 15-3; CEA: carcinoembryonic antigen; CA-125: cancer antigen 125; TSGF: malignant tumor-specific growth factor; ER: estrogen receptor; PR: progesterone receptor; HER2/neu: human epidermal growth factor receptortype2; VEGF: vascular endothelial growth factor; BMI: body mass index.

Advanced breast cancer stage was also associated with increased serum/nipple discharge levels of the four biomarkers. Patients with lymph node metastases showed increased serum/nipple discharge concentrations of the four biomarkers, and lymph node metastasis was positively correlated with increased nipple discharge concentrations of the four biomarkers. Patients with distant metastases also showed higher levels of nipple discharge biomarkers. In addition, the status of ER, PR and HER2/neu expression in tumor tissue was also reflected in the serum/nipple discharge levels of the evaluated biomarkers. In ER-positive patients, the serum/nipple discharge levels of the four biomarkers were decreased, whereas higher serum/nipple discharge levels were observed in PR-negative patients. In addition, for patients showing HER2/neu expression in tumor tissue, the serum/nipple discharge levels of the four biomarkers were increased. The serum/nipple discharge concentrations of the four biomarkers were influenced by factors such as VEGF expression and the Ki-67 proliferation index in tumor tissue. VEGF, which promotes angiogenesis and the proliferation of endothelial cells, also exerts an important effect on the genesis, development, metastasis, and recurrence of various tumors. Due to the intimate association between the genesis, development, metastasis, and infiltration of breast cancer, serum/nipple discharge levels serve as an important indicator for evaluating the metastasis and infiltration of breast cancer in the clinical setting. A significant difference in the serum/nipple discharge levels of the tested biomarkers was observed between HER2/neu-positive patients and HER2/neu-negative patients, as well as between patients with a Ki-67proliferation index ≤14% and patients with a Ki-67 proliferation index >14% (*p* < 0.05). However, no correlation was noted between the serum/nipple discharge levels of the four biomarkers and BMI, tumor size, onset age, and menarche and menopause age.

### 2.4. The Relationship between Prognosis and Immunohistochemistry Staining Results according to ER, PR, and HER2/neu Status

**Figure 3:**
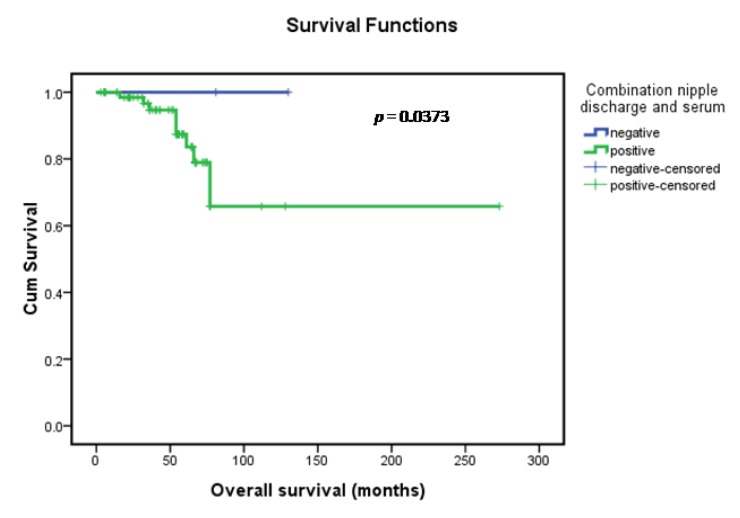
Kaplan-Meier overall survival curves of the patient population under study.

Clinical and pathological information documented at the time of surgery included the clinical stage of the cancer and grade and histology of the tumor. The menopausal status was also documented, and the response to chemotherapy was monitored according to the clinicopathological variables documented at the time of surgical excision and the outcome (progression-free survival (PFS), overall survival (OS)) monitored over a median interval of 62 months. All of the patients were treated with either breast-conserving surgery (*n* = 21) or modified radical mastectomy (*n* = 65), including axillary lymph node dissection (at least 15 nodes resected). Clinical and pathological information documented at the time of surgery included clinical stage of the cancer, grade and histology of the tumor, and amount of the remaining tumor. Patients were monitored for survival and disease progression (no apparent progression or progression) for a median duration of 62 months (range, 3–78 months). Follow-up information was available for 86 of the patients. Seventeen (19.8%) of these patients relapsed and nine (10.5%) died during the course of the follow-up period. Efficacy assessments were performed at 6-week intervals. Progressive disease and stable disease were assessed after the start of adjuvant treatment, and treatment response and disease progression were investigated according to the modified Response Evaluation Criteria in Solid Tumors (RECIST version 1.0) criteria [28]. A complete response (CR) was recorded when the tumor had disappeared completely; a partial response (PR) was recorded when the tumor shrank by more than 30% of the largest diameter; any response was recorded for any degree of response or a decreased size without mention of the tumor dimension; stable disease (SD) was recorded in cases with no sign of recurrent disease within 6 months or change in the tumor size; and progressive disease (PD) was recorded when there was any degree of tumor size increase. PFS was calculated from the administration date of the study drug until PD or death from any cause. Because of the possibility that some patients with no evidence of disease may have artificially influenced the PFS, we checked for the heterogeneity in PFS among patients grouped by their treatment before entering the study (no local treatment *vs.* surgical or local radiotherapy *vs.* whole-breast radiotherapy). Among 86 evaluable patients 58 patients achieved SD, 28 patients achieved PD, and 55 patients experienced some degree of radiologic improvement. In all patients, the overall response rate was 37.2%, with a median PFS of 23 months (95% CI = 11.3–26.0) and a median OS of 62 months (95% CI = 42.7–67.2). The impact of serum/nipple discharge biomarkers, other clinicopathological variables and age on overall survival was presented in Table 5. In the univariate analysis, the nipple discharge concentrations of the patients for whom the four-biomarker combination was positive showed a significantly increased risk of disease progression (hazard ratio = 1.67) and death (hazard ratio = 1.73) (*p* < 0.05) (data not shown in Table 5). In the univariate and multivariate analysis, patients for whom the four-biomarker combination (nipple discharge + serum) was positive showed a significantly increased risk of disease progression (hazard ratio = 1.66) (data not shown in Table 5) and death (hazard ratio = 1.76) (*p* < 0.05). Kaplan-Meier survival curves demonstrated survival differences between the combination (nipple discharge + serum)-positive and -negative patients (*p* = 0.0373) (Figure 4). As shown in Figure 3, the probabilities of OS was lower in combination (nipple discharge + serum)-positive patients than in combination-negative patients. In univariate analysis, nipple discharge biomarkers combination-positive patients had a significantly increased risk of disease progression (hazard ratio = 1.71) (data not shown in Table 5) and death (hazard ratio = 1.418) (*p* = 0.018). The adverse effects of nipple discharge biomarker combination positivity on PFS and OS were lost in the multivariate analysis. When survival outcomes were adjusted for other clinicopathological variables, tumor grade also lost its univariate prognostic significance in the multivariate analysis; only disease stage and combination after surgery maintained their independent effects on overall survival outcome in the multivariate analysis. According to the revised RECIST classification (edition 1.1, 2009) [28], the therapeutic effect was divided into stable disease (SD) and progressive disease (PD), and the effectiveness rate was calculated based on the SD patients. ER and/or PR positivity was considered combined ER and PR positivity, while combined ER and PR negativity was considered both ER and PR negativity.

**Table 5:** Univariate and multivariate overall survival analysis of clinicopathological data.

Variable	Univariate			Multivariate		
	HR ^a^	95% CI ^b^	*p* value	HR ^a^	95% CI ^b^	*p* value
Age	0.945	0.890–1.097	0.079	0.945	0.899–0.993	0.113
Tumor size	0.476	0.219–0.836	0.682	0.528	0.327–2.073	0.598
Lymph node status	0.369	0.175–0.794	0.008	0.762	0.275–2.197	0.049
Histologic grade	0.422	0.374–0.510	0.001	0.592	0.117–2.013	0.067
Disease stage	1.432	0.789–2.876	0.021	1.673	1.016–2.942	0.032
ER & PR status	1.231	0.598–2.763	0.465	1.234	0.465–3.279	0.659
HER2/neu status	1.989	1.386–2.785	0.001	1.592	1.076–3.118	0.072
Serum						
CA153	0.959	0.835–1.101	0.549	0.353	0.114–1.095	0.071
TSGF	1.000	0.974–1.027	0.996	1.024	0.962–1.091	0.449
CA125	0.985	0.903–1.033	0.312	1.202	0.867–1.666	0.270
CEA	1.033	0.959–1.112	1.394	1.492	0.958–2.323	0.076
Combination serum	1.112	0.998–1.102	0.142	1.108	0.798–1.423	0.621
Nipple discharge						
CA153	1.262	1.066–1.493	0.007	4.911	0.925–16.084	0.062
TSGF	1.558	0.774–2.332	0.254	2.834	0.962–4.231	0.317
CA125	1.019	0.971–1.069	0.441	0.741	0.499–1.100	0.137
CEA (ng/mL)	1.059	0.966–1.160	0.224	1.396	0.943–2.067	0.095
Combination ND	1.418	0.838–2.319	0.018	1.562	0.732–3.442	0.263
Combination serum/ND	1.760	1.083–2.597	0.015	1.879	1.091–3.214	0.021

^a^ Hazard ratio (HR) estimated from Cox proportional hazard regression model; ^b^ Confidence interval (CI) of the estimated HR.CA15-3: cancer antigen 15-3; CEA: carcinoembryonic antigen; CA-125: cancer antigen 125; TSGF: malignant tumor-specific growth factor; CI: confidence interval; ND: nipple discharge.

The negative rate of ER and PR expression in the breast cancer observation group was 16.28% (14/86), whereas the positive rate of ER and/or PR expression was 73.72% (72/86). The follow-up results for all patients demonstrated that the effectiveness rate of 14 patients with negative ER and PR expression was 35.71% (5/14), in which PD and SD occurred in 9 and 5 cases, respectively. Among the remaining patients, the effectiveness rate was 73.61% (53/72), in which PD and SD occurred in 19 and 53 cases, respectively (Table 6). HER2/neu expression was positive in 28 (32.56%) patients in the breast cancer group, whereas negative expression was observedin 58 (67.44%) patients. The follow-up results demonstrated that the effectiveness rate of 28 patients with positive HER2/neu expression was 32.14% (9/28), in which PD and SD occurred in 19 and 9 cases, respectively. Among the patients with HER2/neu-negative expression, the effectiveness rate was 84.48% (49/58), in which PD and SD occurred in 9 and 49 cases, respectively. Thus, the expression of breast cancer receptors and HER2/neu was reasonably associated with the prognosis of patients (Table 7).

**Table 6:** Relationship between prognosis and ER & PR status.

ER & PR status	SD	PD	*p* value
ER & PR negative	5	9	<0.001
ER & PR positive	53	19	

χ^2^ test; χ^2^ = 7.67; SD: stable disease; PD: progressive disease.

**Table 7:** Relationship between ER & PR expression and SD and PD.

ER & PR status	SD	PD	*p* value
ER & PR negative	49	9	<0.001
ER & PR positive	9	19	

χ^2^ test; χ^2^ = 23.56; SD: stable disease; PD: progressive disease.

## 3. Materials and Methods

### 3.1. Ethical Statement

The present study was approved by the ethical committee at Rizhao People’s Hospital on 16 February 2006 (No. RZPH2006012). Written informed consent was obtained from all of the patients before their participation in the current study.

### 3.2. Patients

A series of 282 Chinese females treated at Rizhao People’s Hospital were enrolled in the present study from June 2000 to June 2013. Among these patients, 86 had breast cancer, 136 had benign breast lesions, and 60 were normal pregnant women with no evidence of malignancy or a familial history of breast cancer. The diagnosis was verified by histological methods, and pathological categorization was determined according to the current WHO classification system (2012). All of the patients signed informed consent forms, and the current study was approved by the local ethical committee. In the observation group, all of the patients were treated with either breast-conserving surgery (*n* = 21) or modified radical mastectomy (*n* = 65), including axillary lymph node dissection (at least 15 nodes resected). Clinical and pathological information documented at the time of surgery included the clinical stage of the cancer, grade and histology of the tumor, and amount of the remaining tumor. Tumors were staged according to AJCC criteria [23]. Histologic classification was based on the WHO classification system and modified Bloom-Richardson score. Patients were monitored for survival and disease progression (no apparent progression or progression) for a median duration of 62 months (range, 3–78 months). Follow-up information was available for 86 of the patients. Seventeen (19.8%) of these patients relapsed, and nine (10.5%) died during the course of the follow-up period. The patients ranged in age from 21 to 78 years, and the mean age was 47.5 ± 5.3 years. The patients’ body weight ranged from 42 to 79 kg, and the mean body weight was 55.8 ± 4.7 kg. No patient was treated with radiotherapy, chemotherapy, or endocrine therapy before surgery. In the benign breast lesion group, the patient ages ranged from 22 to 74 years, and the mean age was 46.2 ± 5.1 years. The patients’ body weight ranged from 42 to 73 kg, and the mean body weight was 55.3 ± 6.7 kg. No significant difference was noted regarding general data such as gender, age, and body weight, indicating good comparability between the observation group and benign breast lesion group (*p* > 0.05). Data concerning ER, PR, HER2/neu, and VEGF status, Ki-67 proliferation index, and histological grade (including pathological tumor properties) were recorded. Patients assessed for the expression of ER, PR, HER2/neu, and VEGF were grouped as positive or negative. Regarding the Ki-67 proliferation index, patients were divided into two groups: those with a Ki-67 proliferation index between 1% and 14% and those with a Ki-67 proliferation index >14%. Concerning the histological grade, patients were classified into two groups: grade II and grade III. Regarding the levels of CA15-3, CA125, CEA, and TSGF, the patients were divided into two groups: those with a normal level or high level in peripheral blood or nipple discharge. The follow-up duration ranged from 3 months to 2 years, and the levels of the indicators mentioned above were detected through dynamic blood draws.

### 3.3. Measurement of Biomarkers in Patient Serum and Nipple Discharge Samples

For CA15-3, TSGF, CA125, and CEA analysis, 3 mL of heparinized blood and 0.5 mL of nipple discharge were drawn from each individual. The biomarkers were detected using electrochemiluminescence methods in the clinical laboratory at Rizhao People’s Hospital, and the results were compared to those of 60 normal pregnant women. Youden’s index was also used to determine the CA15-3, TSGF, CA125, and CEA serum and nippledischarge marker cut-off values. The cut-off values for CA15-3, TSGF, CA125, and CEA in serum were 25.00, 70.00, 35.00 U/mL, and 3.40 ng/mL, respectively, and those in nipple discharge were 35.00, 95.00, 40.00 U/mL, and 9.8 ng/mL, respectively. In the present study, the CA15-3, TSGF, CA125, and CEA levels in most of the patients were above the normal range when surgery was performed using conventional methods, and the values were also consistent with those previously reported in the literature.

### 3.4. Pathologic Analysis

Breast tissue samples were fixed in 10% neutral buffered formalin and embedded in paraffin at 4 °C for 24 h. Tissue sections at 5-μm thickness were deparaffinized and rehydrated using standard procedures. The specimens were examined under a binocular-dissecting microscope. The pathological diagnosis was independently verified by two pathologistsusing histological methods, and pathological categorization was determined according to the current WHO classification system. The immune histochemical UltraSensitive™ S-P method was employed to detect the expression of ER, PR, HER2/neu, VEGF, and the Ki-67 index. The pathologists were blinded to the subject’s clinical history and the results of the immunohistochemistry staining assay. The pathological reading was determined for each biopsy slide with an overall pathological diagnosis determined for each subject. The tumor grade was determined according to the modified Bloom-Richardson score. The grade was obtained by summing the scores for tubule formation, nuclear pleomorphism, and mitotic count, which were scaled as 1, 2, or 3. The final scores ranged between 3 and 9 and were then divided into three grades (I–III). The final grading scores were as follows: sum of points, 3–5, final grade I; 6–7, II; and 8–9, III.

Immunoreactions were processed using the UltraSensitive™ S-P Kit (Maixin-Bio, Fuzhou, Fujian, China) according to the manufacturer’s instructions, and signals were visualized using the Diaminobenzidine (DAB) substrate, which stains the target protein yellow. Negative controls were used in which the primary antibody was replaced with PBS containing 0.1% bovine serum albumin at the same concentration as the primary antibody. The positive controls consisted of tissues known to express the antigen being studied. The immunoreactivity of ER, PR, or Ki-67 was expressed as the percentage of cancer cells showing nuclear reactivity. The immunoreactive score (IRS) was obtained by multiplying the percentage of positive cells and the staining intensity. Briefly, a proportion score was assigned that represented the estimated proportion of positive tumor cells on the entire slide. For each histological section, the percentage of positive cells was scored as 0 (<5%), 1 (6%–25%), 2 (26%–50%), 3 (51%–75%), and 4 (>75%), and the staining intensity was scored as 0 (negative), 1 (weak), 2 (moderate), and 3 (strong). Immunohistochemical results with an IRS of 0 were considered negative, an IRS of 1–4 was considered weakly positive, an IRS of 5–8 was considered moderately positive, and an IRS of 9–12 was considered strongly positive. For ER, PR, and Ki-67 expression, the percentage of cancer cells showing nuclear reactivity was recorded after inspection of all of optical fields at ×200, and the mean value was used to score each case. Tumors showing expression in >1% of cancer cells were considered positive. ER and/or PR positivity was considered combined ER and PR positivity, and combined ER and PR negativity was considered both ER and PR negativity. For the Ki-67 proliferation index, patients were divided into two groups: those with a value between 1% and 14% and those with a value >14%. In addition, regarding the cell membrane reactivity of HER2/neu, oncoprotein expression was evaluated following a similar approach, and the mean value was used to score each case. Tumors expressing HER2/neu in >30% of the cancer cells were considered to show positive expression. VEGF was localized in the cytoplasm and the membrane. Cells were classified according to the positive rate and color intensity as follows: negative, number of positive cells <25%; positive, brown particles, number of positive cells ≥25%.

For analyses, we considered the age at diagnosis, menopausal status, tumor size, histological grading, lymph node involvement, stage, status of ER, PR, HER2/neu, and VEGF expression, and the Ki-67 indexin relation to the level of the four tumor markers in the serum and nipple discharge. Among the 86 breast cancer patients, 44 had lymphnode-negative disease, and 42 had lymphnode-positive disease; in addition, 17 had distal metastasis-positive disease 69 had distal metastasis-negative disease, and 17 patients had recurrence.

### 3.5. Statistical Analysis

SPSS version 17.0 statistical software (SPSS Inc.: Chicago, IL, USA) was used to analyze the data, and the results were expressed as the mean and standard deviation (SD). Because the distribution of the four breast cancer markers, which were measured in serumand nipple discharge tumor extracts, was not Gaussian, the nonparametric Mann-Whitney U-test was used to determine differences between the benign and malignant groups, and the nonparametric Kruskal-Wallis test was used for the analysis of differences among more than two groups. The relationship of this dichotomous variable to other clinicopathological correlates was established using chi-squared (χ^2^) or Fisher’s exacttests, as appropriate. ROC curves were generated based on comparisons with the control group. The cut-off values were selected using ROC curves as the reference, based on high sensitivity or high specificity. The impact of the serumand nipple discharge biomarker levelon patient survival and PFS was assessed using hazard ratios calculated by both univariate and multivariate Cox proportional hazards regression models according to the methods of Xie *et al.* [29]. In the multivariate analysis, the clinical and pathological variables affecting survival, including stage of disease, tumor grade, histologic type, and age, were adjusted. Kaplan-Meier OS curves was constructed to demonstrate the survival differences between the biomarker level-positive and biomarker level-negative patients. The log-rank test was used to examine the significance of the differences among the survival curves. We considered the age at diagnosis, menopausal status, tumor size, histological grading, lymph node involvement, status of ER, PR, HER2/neu, and VEGF expression, and Ki-67 index in relation to the level of the four tumor markers in serum and nipple discharge. The impact of the tumor biomarker level (positive or negative) on survival and disease progression was determined by univariate and multivariate models for each of the subgroups. A *p* value less than 0.05 was deemed statistically significant.

## 4. Conclusions

In the present study, we analyzed the CA15-3, CA125, CEA, and TSGF levels in nipple discharge and serum samples from women with and without breast cancer as well as women with galactophore hyperplasia, and breast cancer post-surgery. The nipple discharge and serum levels of the four biomarkers in breast carcinoma patients were significantly higher than those in normal women and women with benign breast disease. Additionally, the levels of CA15-3, CA125, CEA, and TSGF in nipple discharge were significantly higher than those in serum, and these levels showed a positive correlation with the Ki-67 index, tumor grade, clinical stage, lymph node metastasis, and tumor recurrence and a negative correlation with the level of ER and PR expression. The sensitivity of the combined detection of the four biomarkers in both nipple discharge and serum was significantly higher than that using other detection methods. The follow-up duration and levels the combined tumor markers in both nipple discharge and serum samples were helpful for the stratification of preoperative patients, and this four tumor marker panel may therefore serve as an early strategy for monitoring recurrence, metastasis, and clinical staging of tumors in the clinical setting; however, this approach did not increase the sensitivity of detecting patients with early breast cancer. Although a significant amount of additional work should focus on CA15-3, CA125, CEA, and TSGF, these results suggest that the CA15-3, CA125, CEA, and TSGF levels in nipple discharge may serve as novel biomarkers for breast cancer. In addition, our work suggests that CA15-3, CA125, CEA, and TSGF may be detected in nipple discharge as well as other breast lesion samples. Therefore, related validation work should continue in further analyses. Univariate analysis revealed that combined detection of CA15-3, CA125, CEA, and TSGF in nipple discharge served as novel biomarkers for the diagnosis and prognosis of breast cancer. Multivariate analysis revealed that the positivity of the combined detection of the four biomarkers in both nipple discharge and serum was significantly higher than the positivity of other detection methods, and the panel of the four biomarkers was retained as an independent prognostic variable in breast cancer patients.

## Abbreviations

CA15-3: cancer antigen 15-3
CEA: carcinoembryonic antigen
CA125: cancer antigen 125
TSGF: malignant tumor-specific growth factor
ER: estrogen receptor
PR: progestrone receptor
HER2/neu: human epidermal growth factor receptor type 2
VEGF: vascular endothelial growth factor
ND: Nipple Discharge
HR: Hazard ratio
SD: standard deviation
PD: progressive disease
PFS: Progression-free survival
OS: overall survival
CI: confidence interval
RECIST: Response Evaluation Criteria in Solid Tumors
BMI: body mass index
IQR: inter-quartile range
ROC: receiver operating characteristic
AUC: area under the curve
FDA: Food and Drug Administration
DCIS: ductal carcinoma *in situ*
DAB: Diaminobenzidine
